# Early uptake and continuous accumulation of thallium-201 chloride in a benign mixed tumor of soft tissue: Case Report

**DOI:** 10.1186/1746-1596-5-34

**Published:** 2010-05-30

**Authors:** Shigenori Nagata, Yu-Fen Jin, Katsuhiko Yoshizato, Masanori Kitamura, Norishige Iizuka, Misa Song, Miki Tomoeda, Michiko Yuki, Chiaki Kubo, Hidenori Yoshizawa, Hidetatsu Outani, Kenichiro Hamada, Nobuhito Araki, Masahiro Funauchi, Yasuhiko Tomita

**Affiliations:** 1 Department of Pathology, Osaka Medical Center for Cancer and Cardiovascular Diseases, Higashinari, Osaka 537-8511, Japan; 2 Department of Orthopedics and Osaka Medical Center for Cancer and Cardiovascular Diseases, Higashinari, Osaka 537-8511, Japan; 3 Department of Nuclear Medicine, Osaka Medical Center for Cancer and Cardiovascular Diseases, Higashinari, Osaka 537-8511, Japan

## Abstract

A case of benign mixed tumor of the soft tissue in a 64-year-old Japanese male is presented. He noticed a painless, elastic hard mass sized 3 cm in the right knee, which gradually grew larger and harder in the last 5 years. Magnetic resonance imaging demonstrated a mass lesion embedded in the subcutaneous tissue with low and high signal intensity at T1- and T2-weighted images, respectively. Tl-201 scintigraphy showed an early uptake of Tl-201 within the lesion at 10 minutes after injection, which was slightly decreased but still continued at 2 hours later. The patient underwent a resection of tumor, and the pathological diagnosis was a benign mixed tumor of soft tissue without high vascularity, characterized by histological features similar to pleomorphic adenomas in the salivary glands. Immunohistochemical study proved expression of Na^+^/K^+^-ATPase of tumor cells. Overexpression of Na^+^/K^+^-ATPase of the tumor might be responsible for the early uptake of Tl-201, and poor vascular structure in this tumor might lead to continuous accumulation. The Tl-201 scintigraphic features of mixed tumor of soft tissue are assessed to resemble those of malignant soft tissue tumors.

## Background

Mixed tumors can occur in soft tissue as well as in the skin and soft palate [1,2], although only 18 cases have been described to be primary in soft tissue [3,4]. Mixed tumors of soft tissue demonstrate similar histological features to pleomorphic adenomas of the salivary glands, having both benign and malignant forms in the same way. The feature of thallium-201 chloride (Tl-201) scintigraphy in mixed tumors of soft tissue is still unknown. Herein, we present a case of benign mixed tumor arising in the subcutis, which demonstrated an early uptake of Tl-201 with slightly decreased accumulation in the delayed scan. Immunohistochemical study proved the tumor cells to express sodium/potassium (Na^+^/K^+^)-ATPase, which is considered to play an important role in uptake and accumulation of Tl-201 [5-8].

## Case Presentation

**Figure 1:**
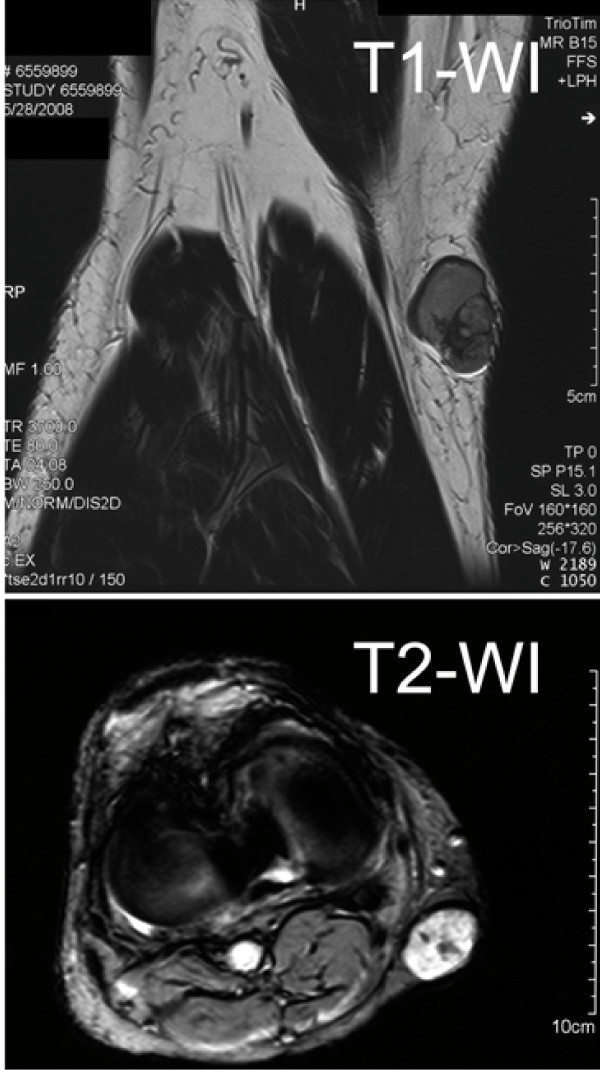
**MRI showing a mass lesion embedded in the subcutaneous tissue on the medial posterior side of the right knee, demonstrating low and high signal intensity in T1- and T2-weighted images (arrows in upper and lower), respectively**.

**Figure 2:**
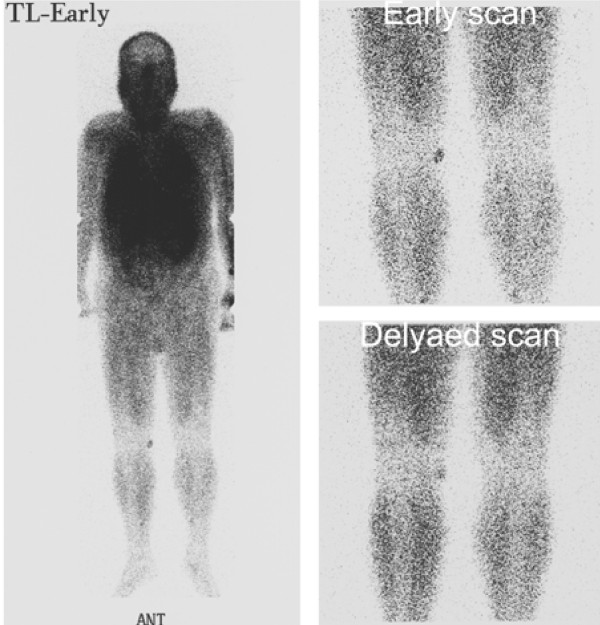
**Tl-201 scintigraphy of the whole body at the early phase (left)**. Tl-201 was uptaken into the tumor in the right knee at the early scan (right upper) and continuously accumulated at the delayed scan (right lower).

A 64-year-old Japanese male, who had no past medical history or present systemic disease, noticed a mass in the right knee 10 years ago. He had no history of a pre-ceding trauma in the lesion. The mass gradually grew larger and harder in the last 5 years, accompanied by sense of tension in a sitting position. Physical examination revealed a smooth, elastic hard, and mobile mass of 3 cm in the largest diameter in the medial posterior side of his knee, which was painless and caused no neurological abnormality. Difficulty in physical activities such as deep knee bend and square-sitting was observed. Computed tomography scan revealed a mass lesion of heterogeneous density with a cystic area, enhanced not in the early arterial phase but in the delayed phase. Magnetic resonance imaging demonstrated a mass lesion embedded in the subcutaneous tissue with low and high signal intensity at T1- and T2-weigthed images, respectively (Fig. 1). Thallium-201 chloride (Tl-201) scintigraphy showed an uptake of Tl-201 into the mass at early scan at 10 minutes after injection, which slightly decreased but still accumulated at 2 hours later (Fig. 2). The patient underwent a resection of tumor for a tentative diagnosis of neurilemoma of degenerative type ("ancient Schwannoma"). Surgeons found a tumor encapsulated by a thin fibrous capsule in the subcutis and marginally excised it. The patient was uneventfully discharged from the hospital and free from locally recurrent or metastatic disease two years after surgery.

### Pathological findings

**Figure 3:**
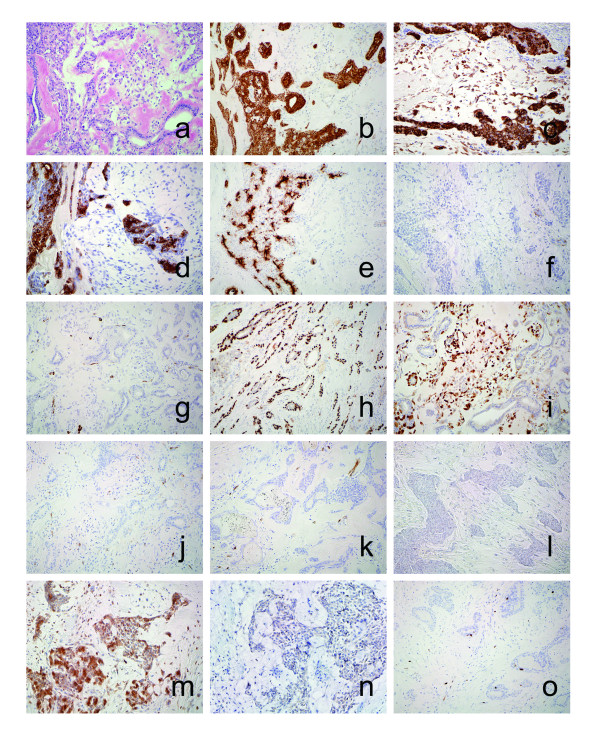
**Comprehensive immunohistochemical staining:** a, representative section by H-E stain (×200); b, Cytokeratin (CK) AE1/AE3 (×200); c, CK CAM5.2 (×200); d, CK 34βE12 (×200); e, EMA (×200); f, desmin (×200); g, α-smooth muscle actin (×200); h, p63 (×200); i, S-100 protein (×200); j, CD31 (×200); k, CD34 (×200); l, p53 (×200); m, bcl-2 (×200); n, p16 (×200); o, Ki-67 (×200).

Grossly, the resected specimen was a white-colored, elastic hard mass measuring 3.0 × 2.0 × 1.5 cm, and on cut-surfaces, consisted of grayish solid area and whitish gelatinous component with a cystic space containing a little amount of serous fluid. Microscopically, uniform-appearing epithelioid cells without cytoplasmic vacuolation were arranged in nests, cords, and ductules, and spindle-shaped myoepithelial cells embedded in abundant chondromyxoid matrix, accompanied by osseous metaplasia (Fig. 3). Neither vascular proliferation nor blood pools were recognized. Mitotic figures and nuclear pleomorphism were absent.

Comprehensive immunohistochemical findings are demonstrated in Figure 3. The epithelial component of the tumor was diffusely stained with cytokeratin (CK) AE1/AE3 and CAM5.2, and partially with CK 34βE12. Epithelial membrane antigen (EMA) was strongly expressed on the luminal surface of glandular structures. Mesenchymal tumor cells were totally negative for these epithelial markers except scattered cells positive for CAM5.2. Desmin and α-smooth muscle actin were not expressed within the tumor. Glandular component was positive for anti-p63, indicating the basal myoepithelial characteristics of the cells. Many mesenchymal tumor cells were stained with anti-S-100 protein. Vascular structures indicated by CD31 and CD34 were scant in the stroma. Bcl2 was strongly expressed in the epithelial component with a weaker intensity in the mesenchymal component. Epithelial tumor cells were faintly stained with anti-p16 in the cytoplasm. Ki-67 labeling index was less than 1% and p53-positive cells were rarely seen in both epithelial and mesenchymal lesion, indicating the low proliferation rate and the absence of disruption of p53 tumor-suppressor pathway. These findings indicated that the tumor to be composed of epithelial and mesenchymal lesions, and the epithelial component having myoepithelial characteristics. The pathological diagnosis was a benign mixed tumor arising in the subcutis of the knee. All the primary antibodies used for the differential diagnosis were from Dako A/S (Glostrup, Denmark) except CAM5.2 (Becton Dickinson, Flanklin Lakes, NJ, USA) and anti-p16 (Novocastra Laboratories Ltd., Newcastle upon Tyne, UK).

**Figure 4:**
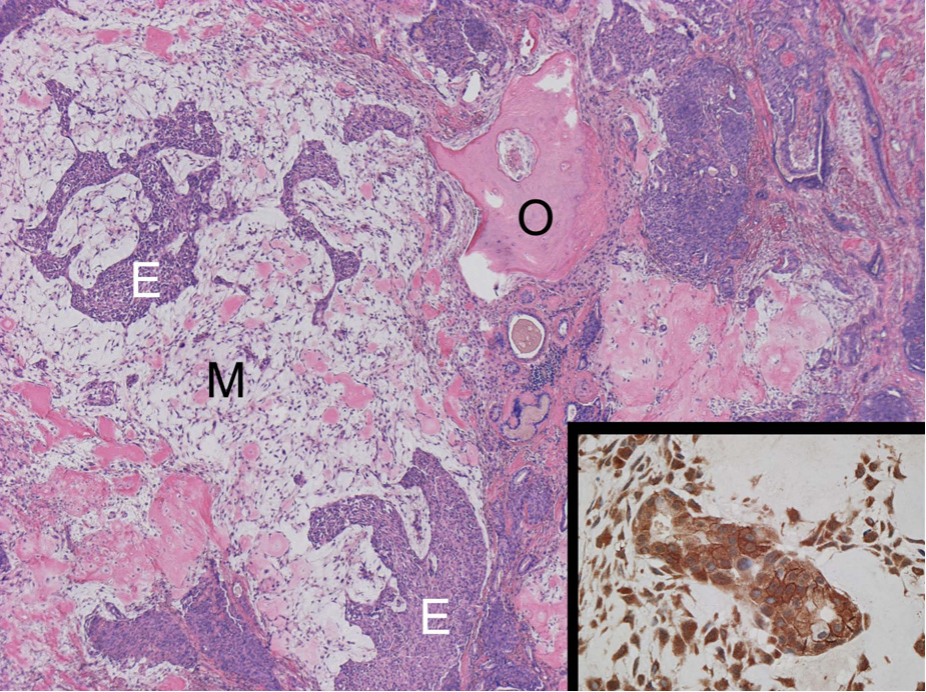
**Histological feature of mixed tumor of soft tissue with a variety of components; epithelial component E, myoepithelial component M, and osseous metaplasia O (H-E stain, ×40)**. Tumor cells overexpressing anti-Na^+^/K^+^-ATPase antibody (small window in right lower corner, immunohistochemical staining, ×400).

Immunohistochemical analysis using anti-mouse monoclonal antibody against Na^+^/K^+^-ATPase α (M7-PB-E9, Affinity BioReagents Inc., Golden, CO, USA) demonstrated an expression of Na^+^/K^+^-ATPase in cell membrane and cytoplasm of tumor cells (Fig. 4).

## Dissucusion

Well-characterized as "pleomorphic adenomas" in salivary glands, mixed tumors may also occur in the skin and soft palate, and have been recently recognized to be primary in soft tissue [1,2]. Microscopically, mixed tumors of soft tissue show similar morphologic feature to their salivary gland counterparts. Mixed tumors, myoepitheliomas, and parachordomas originated from the soft tissue share many clinical and histological features, all of which are considered within the same spectrum. The vast majority of these tumors arise in the subcutaneous or deep subfascial soft tissue of extremities, more frequently in the upper than the lower [9], usually affect adults with a significant number of children [3,9]. Only 18 cases of mixed tumors of soft tissue were reported [4], and 33 percent of those were described to meet histological criteria for malignancy resulting in local recurrence or metastasis [4].

In the present case, diagnostic differential considerations include other benign lesions, such as neurilemoma of degenerative type and organized hematoma that may radiologically demonstrate a granular pattern of low in high signal intensity on T2-weighted MRI images indicating calcification or hemorrhage within the tumor. However, these diagnoses were excluded by the histological finding of tumor cells showing both epithelial and mesenchymal characteristics. Given the morphologic heterogeneity of this tumor, the differential diagnosis is quite broad and depends in large part upon the predominant cell type and stromal component [10,11]. Other candidate was synovial sarcoma with both epithelial and mesenchymal lesion in the tumor expressing bcl-2. Although the present case showed strong bcl2 expression in the cytoplasm, however diagnosis of synovial sarcoma was deniable as the epithelial cells showed myoepithelial marker p63. Metastasis of myoepithelial carcinoma originated from other organs, such as head and neck might be excluded by the benign histology of the tumor.

This is the first report of Tl-201 scintigraphic features in a benign mixed tumor of soft tissue in our reviewing literatures. It is generally considered that Tl-201 uptake around tumor cells mainly depends on the volume of blood flow and blood pools [12]. In the second step, Tl-201 has similar ion-radius and physical effects similar to potassium [12], having five times the affinity to cell as potassium, and is transported into the cell instead of potassium as a result [13]. The preferential transportation of Tl-201 mainly depends on Na^+^/K^+^-ATPase, which is overexpressed in a variety of malignant tumors [5-8] and some benign tumor cells [14]. In the present case, although vascularity of tumor was not high by morphological assessment, imunohistochemical study revealed expression of Na^+^/K^+^-ATPase in most tumor cells. This result may account for a mechanism of the early uptake and continuous accumulation of Tl-201 in a benign mixed tumor of soft tissue.

Conclusive significance of thallium-201 scintigraphy in soft tissue tumors has been still controversial within our knowledge. Positive examination often indicates malignant tumors, but some benign tumors are occasionally estimated as positive [15,16]. Accumulation of Tl-201 at delayed scan has been reported as a more sensitive finding distinguishing malignancies from benignancies [17]. In our case, we considered the continuous accumulation of Tl-201 at delayed scan as a potential sign indicating malignancy, although it was denied by histological examination including immunohistochemistry.

## Conclusions

We accessed Tl-201 scintigraphic features of a mixed tumor of soft tissue showing an early uptake and longtime accumulation of Tl-201. Overexpression of Na^+^/K^+^-ATPase in the tumor was considered to be responsible for the early uptake followed by continuous accumulation of Tl-201 due to poor vascular structure of the tumor. We emphasize that clinicians should include such benign tumor when they find ambiguous Tl-201 scintigraphic feature.

## Consent

Written informed consent was obtained from the patient for publication of this case report and any accompanying images. A copy of the written consent is available for review by the Editor-in-Chief of this journal.

## Competing interests

The authors declare that they have no competing interests.

## Authors' contributions

SN participated in the pathological examination of the case, the design of the study and drafting the manuscript. MK, NI, MS, MY, CK, and HY participated in the pathological examination of the case. YFJ, KY, and MT participated in the immunohistochemical analysis. HO, KH, and NA participated in collecting clinical data and images. MF participated in the radiological analysis. YT participated in its design and coordination and helped to draft the manuscript.
